# A Two-Stage Reinforcement Learning Framework for Humanoid Robot Sitting and Standing-Up

**DOI:** 10.3390/biomimetics10110783

**Published:** 2025-11-17

**Authors:** Xisheng Jiang, Shihai Zhao, Yudi Zhu, Qingdu Li, Jianwei Zhang

**Affiliations:** 1 School of Optoelectronic Information and Computer Engineering, University of Shanghai for Science and Technology, Shanghai 200093, China; 221240064@st.usst.edu.cn (X.J.); 242260504@st.usst.edu.cn (S.Z.); 221240082@st.usst.edu.cn (Y.Z.); 2 Institute of Machine Intelligence, University of Shanghai for Science and Technology, Shanghai 200093, China; 3 Zhongyu Embodied AI Laboratory, Zhengzhou 450046, China; 4 Shanghai Droid Robotics Co., Ltd., Shanghai 200082, China; 5 Department of Informatics, University of Hamburg, 20146 Hamburg, Germany

**Keywords:** reinforcement learning, two-stage, humanoid robots, bi-level optimization

## Abstract

In human daily-life scenarios, humanoid robots need not only to stand up smoothly but also to autonomously sit down for rest, energy management, and interaction. This capability is crucial for enhancing their autonomy and practicality. However, both sitting and standing involve complex dynamics constraints, diverse initial postures, and unstructured terrains, which make traditional hand-crafted controllers insufficient for multi-scenario demands. Reinforcement Learning (RL), with its generalization ability across high-dimensional state spaces and complex tasks, offers a promising solution for automatically generating motion control policies. Nevertheless, policies trained directly with RL often produce abrupt motions, making it difficult to balance smoothness and stability. To address these challenges, we propose a **two-stage reinforcement learning framework**: In the first stage, we focus on exploration and train initial policies for both sitting and standing, with relatively weak constraints on smoothness and joint safety, and without introducing noise. In the second stage, we refine the policies by tracking the motion trajectories obtained in the first stage, aiming for smoother transitions. We model the tracking problem as a bi-level optimization, where the tracking precision is dynamically adjusted based on the current tracking error, forming an adaptive curriculum mechanism. We apply this framework to a 1.7 m adult-scale humanoid robot, achieving stable execution in two representative real-world scenarios: sitting down onto a chair, stand up from a chair. Our approach provides a new perspective for the practical deployment of humanoid robots in real-world scenarios.

## 1. Introduction

Humanoid robots, due to their human-like body structure and motion characteristics, have long been a focus of research, with continuous efforts aimed at accelerating their integration into real-world environments. Thanks to advances in hardware and control methods, significant progress has been made in bipedal gait control [1,2,3,4] and dual-arm manipulation [5,6,7,8]. However, one of the most fundamental yet crucial capabilities—sitting and standing-up control—has been largely overlooked in existing studies. This is primarily because most humanoid robots are assumed to operate from an already-standing initial posture.

The lack of sitting and standing-up skills imposes notable limitations on real-world applications: robots cannot naturally transition from a seated (resting) posture to a standing (working) state as humans do. Mastering these capabilities would greatly enhance the autonomy and applicability of humanoid robots in everyday scenarios. Motivated by this, we investigate how humanoid robots can learn to perform sitting and standing-up tasks in real-world environments. However, we observe that standing-up problems differ from typical motion control tasks in three key aspects, making the direct application of existing methods insufficient.

Non-periodic behavior. Unlike walking, which exhibits a clear periodic pattern with alternating leg movements, the sit-stand process does not follow a stable periodic pattern. The robot must autonomously explore an effective sequence of contacts to complete the sit-stand transition, which significantly increases the difficulty of optimization. Moreover, common “phase coupling” techniques used in walking control (e.g., synchronization between left and right legs) completely fail in sit-stand tasks.

Diverse contact modes. Unlike typical locomotion tasks, the sit-stand process involves contacts that are not limited to the legs. In fact, multiple parts of the robot’s body may already be in contact with the ground, and the robot needs to actively use these body parts to interact with the environment to complete the transition. Therefore, common simplifications in walking tasks (e.g., freezing or decoupling the upper body, coarsely modeling upper-body collisions, or using large simulation time steps) are no longer applicable in sit-stand tasks.

Reward sparsity. Compared with locomotion or manipulation tasks, designing reward functions for the sit-stand behavior is more challenging. In walking tasks, dense reward feedback can be obtained through velocity tracking, where the robot receives clear feedback on whether it follows the commanded velocity within tens of simulation steps. However, during the sit-stand process, many initial movements may appear “negative.” For example, the torso may need to press down for a few seconds before finally lifting up, leading to sparse and delayed reward signals, which significantly increases the difficulty of learning.

We propose a **two-stage reinforcement learning (RL) framework** to address the challenges of non-periodic contacts, diverse contact modes, and sparse rewards in sit-stand control. Owing to its capability in handling transient and highly non-linear dynamics, the proposed framework can be readily extended to dynamic locomotion tasks such as walking and running, and further generalized to other types of mobile robots.

**Stage 1: action Discovery.** In the first stage, we train the robot in a simplified environment (no noise, no randomization, and weak constraints) to discover feasible sit-stand motion sequences. Due to the sparsity and ambiguity of the reward signal, this stage focuses on identifying a valid trajectory for completing the sit-stand transition. To promote natural and coordinated motion exploration, we introduce a vertical assistive force during the early training phase of the supine-to-stand task, which helps the robot explore effective strategies.

**Stage 2: action Refinement.** In the second stage, the discovered motion is quantitatively refined using trajectory tracking as a reference. Stronger constraints on action smoothness, joint velocity, and torque are introduced, together with noise and domain randomization. We design an adaptive tracking factor that dynamically modulates the tracking precision according to the current tracking error, yielding a smoother and more hardware-ready control policy.

We incorporate multi-level curriculum learning strategies within each stage, including:

(1) During stage 1, the vertical assistive force and action amplitude are gradually adjusted based on the robot’s center-of-mass height.
(2) From stage 1 to stage 2, we adopt a progressive training schedule: standard terrains → random terrains, fixed initial poses → random initial poses, and no noise/randomization → with noise and domain randomization.

**Figure 1:**
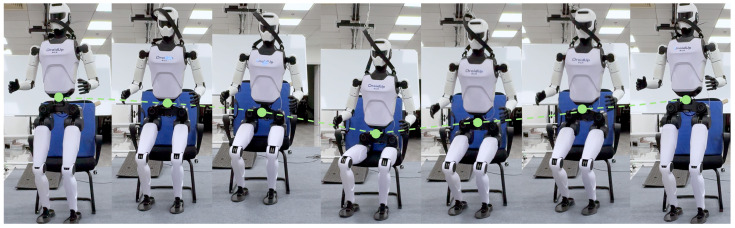
Snapshots of real robot sitting and standing motion. The dashed line indicates the trajectory of the robot’s center of mass.

We validate our framework on a cable-driven humanoid robot X02 in both simulation and real-world experiments. In the real world, X02 successfully performs smooth sit-down and stand-up on various terrains, exhibiting excellent smoothness, stability, and robustness to external disturbances. Figure 1 illustrates our controller enabling the real robot to complete the sitting and standing-up process. (see demonstration video at https://youtube.com/shorts/6iN3a6X3-LM (accessed on 21 October 2025)).

Our main contributions are summarized as follows:

1. We propose a two-stage RL framework that decouples action discovery from action refinement, effectively addressing the challenges of sparse and delayed rewards in sit-stand control.
2. We design a self-adaptive curriculum learning mechanism, which dynamically adjusts trajectory tracking precision and progressively introduces terrain randomization and noise, improving generalization and real-world robustness.
3. We demonstrate an adult-scale humanoid robot can perform both sit-down and stand-up tasks in the real world using a learned policy, showcasing superior smoothness, stability, and disturbance resistance.

The structure of this paper is as follows. Section 2 reviews related work in robotic control, particularly for sit-to-stand motions. Section 3 introduces the proposed two-stage learning framework and the Adaptive Motion Tracking mechanism. Section 4 and Section 5 present simulation and real-world experiments demonstrating the effectiveness of the framework on sit-down and stand-up tasks.

## 2. Related Work

### 2.1. Control Methods for Humanoid Robots

Humanoid robots, owing to their human-like body structure, high degrees of freedom, and strong natural affinity for human interaction, have long been a central research topic in robotics. Early studies primarily relied on model-based control algorithms, such as the Zero Moment Point (ZMP) principle, trajectory optimization, and Model Predictive Control (MPC). It is worth noting that Generalized Predictive Control (GPC) also represents a critical issue in the control of walking robots and complex dynamic systems. These approaches have yielded significant advancements in essential locomotion behaviors, such as bipedal walking, dynamic running, and leaping motions.

However, their generalization ability is limited when applied to complex or unknown environments. In particular, for whole-body coordination tasks such as sit-to-stand motions, traditional approaches often rely on manually designed motion trajectories combined with optimization techniques [9,10,11,12]. While effective in simulation, their practical application is restricted by several factors:

(1) High computational cost, making real-time execution challenging; (2) Sensitivity to external disturbances, resulting in poor robustness [13,14]; (3) Heavy dependence on accurate dynamic modeling and actuator parameters [15].

Therefore, in highly dynamic, multi-contact, and uncertain real-world environments, these traditional model-based methods are difficult to extend to complex tasks such as sit-to-stand control.

### 2.2. Reinforcement Learning for Humanoid Robot Control

In recent years, Reinforcement Learning (RL) has shown remarkable performance in high-dimensional control tasks, making it one of the core methods in humanoid robot research. By training policies in high-fidelity physics simulations and deploying them to the real world via Sim-to-Real techniques, RL has achieved significant breakthroughs on a wide range of robotic platforms, including quadrupedal robots [15,16] and humanoid robots [17,18,19,20].

In humanoid robotics, RL has been successfully applied to learn robust locomotion over challenging terrains [1,4], dynamic motions such as jumping and vaulting [21,22], and vision-based dynamic control strategies [3,4], demonstrating strong generalization and adaptability to high-dynamic tasks. Furthermore, by leveraging human motion capture data or imitation learning [23,24,25,26], RL has also been used to synthesize natural human-like motion patterns, such as dancing and daily gait behaviors.

Recent research trends have extended RL towards loco-manipulation tasks, which integrate lower-body locomotion with upper-body manipulation, enabling practical skills such as carrying objects or physical interaction [7,27,28]. However, such tasks are often restricted by the assumption that the robot’s feet remain stably in contact with the ground, resulting in relatively limited contact dynamics and control complexity.

In contrast, motions such as rolling, crawling, and sit-stand transitions involve complex whole-body interactions with the environment, characterized by high dynamics and non-periodic contact patterns. These tasks remain among the most challenging yet underexplored areas of RL for humanoid robots. They require policies capable of adapting to time-varying contact topologies and coordinating multi-phase, multi-skill dynamic motion sequences to achieve smooth and stable execution [9,13].

To address these challenges, this work focuses on sit-stand control for humanoid robots and proposes a whole-body RL framework designed to handle the uncertainties arising from high dynamics and diverse contact transitions. This direction provides new insights and a practical foundation for advancing RL applications in multi-modal and natural-interaction control of humanoid robots.

### 2.3. Related Work on Quadruped Robot Recovery

In recent years, reinforcement learning (RL) combined with Sim2Real (simulation-to-reality) techniques has achieved remarkable progress in the recovery control of quadruped robots, providing valuable insights and technical pathways for standing-up control of humanoid robots. Unlike traditional methods that rely on model-based optimization or predefined motion primitives, learning-based approaches emphasize policy exploration through direct interaction with the environment, demonstrating superior generalization and adaptability.

Lee et al. [15] developed a deep RL approach that allows quadruped robots to autonomously recover from complex fallen postures without the need for manually designed motion sequences. Ji et al. [29] demonstrated the robustness of such policies in natural environments (e.g., snowy or uneven terrains), enabling robots to carry objects under uncertain conditions. Wang et al. [30] further enhanced the adaptability of recovery strategies, allowing robots to reliably stand up even under dynamic disturbances and varying environments.

Moreover, RL has empowered quadruped robots with the ability to switch between diverse postures [14,31,32], which is critical for continuous motion control and task transitions. Unlike traditional approaches that depend on accurate system modeling and handcrafted motion coding, RL policies can autonomously discover optimal action sequences to handle complex nonlinear dynamics and environmental uncertainties.

Although quadruped robots are structurally more stable with a lower center of mass and greater support, the challenges they face in recovery control remain comparable to humanoid robots, particularly regarding diverse initial postures, complex contact transitions, dynamic constraints, and robustness to external perturbations. Therefore, research on quadruped recovery control not only validates the effectiveness of RL+Sim2Real in learning complex behaviors but also provides essential insights into policy design, training paradigms, and transfer mechanisms for humanoid robots. Given that humanoid robots have a higher center of mass, reduced stability, and increased sensitivity to dynamic perturbations, extending these approaches to bipedal systems introduces additional challenges.

### 2.4. Related Work on Humanoid Robot Stand-Up Control

Humanoid robots face significant challenges in autonomous stand-up tasks owing to their extensive degrees of freedom and strict requirements for dynamic stability. Existing research on stand-up control methods can be broadly categorized into two approaches: motion-planning-based methods and reinforcement-learning-based methods.

#### 2.4.1. Motion Planning Based Methods

Traditional approaches typically rely on precise dynamics modeling and trajectory planning, where the stand-up task is decomposed into multiple pre-defined postural phases. Early work by Morimoto and Doya [33] addressed simplified stand-up problems using hierarchical reinforcement learning. Later research extended this by modeling different postures as states in a graph and utilizing trajectory optimization or finite state machines to achieve state transitions [9,10,34,35].

In addition, several studies have leveraged human demonstrations [36] and pose symmetry compression [37] to improve planning efficiency, particularly for small-scale humanoid robots.

Despite their success in simulation, these methods encounter critical limitations during real-world deployment:

- They strongly depend on accurate dynamics modeling and actuator parameters [38];
- High computational cost makes it difficult to meet real-time control requirements;
- Poor generalization to complex terrains and diverse initial postures.

As a result, many commercial humanoid robots still rely on pre-defined trajectory playback for stand-up control [39], which lacks flexibility and robustness.

#### 2.4.2. Reinforcement Learning-Based Methods

Reinforcement Learning (RL) acquires behavioral policies through environment interactions, which greatly reduces dependence on precise dynamics models and pre-defined motion trajectories, while demonstrating strong adaptability and robustness. Current techniques are mainly divided into two exploration paradigms: (1) policy learning guided by reference motions or imitation learning [40,41], and (2) policy learning entirely from scratch [42].

Although RL has achieved successful real-world deployment in quadruped robot stand-up tasks [15,29], its application to humanoid robots remains relatively unexplored. Key challenges include low sample efficiency, limited generalization capability, and significant simulation-to-reality (Sim2Real) discrepancies.

To address these challenges, we propose a two-stage RL framework with the following key features:

- **Trajectory-free learning:** Policies are learned in an end-to-end manner without reliance on pre-defined motion primitives.
- **Pose adaptivity:** Capable of handling diverse initial poses and fall states.
- **Enhanced robustness:** Maintains stable stand-up performance under complex terrains and external disturbances.
- **Zero-shot Sim2Real transfer:** Learned policies can be directly deployed on real humanoid robots without fine-tuning.

Overall, RL provides a promising direction for humanoid stand-up control, with its superior generalization and robustness increasingly positioning it as a core alternative to traditional motion-planning methods. By learning predictive control policies directly from interaction data, RL enables dynamic adaptation to varying initial states and environmental conditions, contrasting sharply with pre-planned, rigid control strategies. This predictive and adaptive nature highlights RL’s unique advantage in managing the complex, unstable dynamics inherent to humanoid robots.

## 3. Method

Our goal is to learn a general sit-stand policy that enables a humanoid robot to perform both sitting down and stand up. We primarily focus on two representative scenarios: (1) sitting down from a standing posture; (2) stand up from a seated posture;

Learning a unified policy for these scenarios is challenging due to the distinct kinematic and dynamic constraints involved, as well as the diverse contact sequences required for different initial poses.

**Figure 2:**
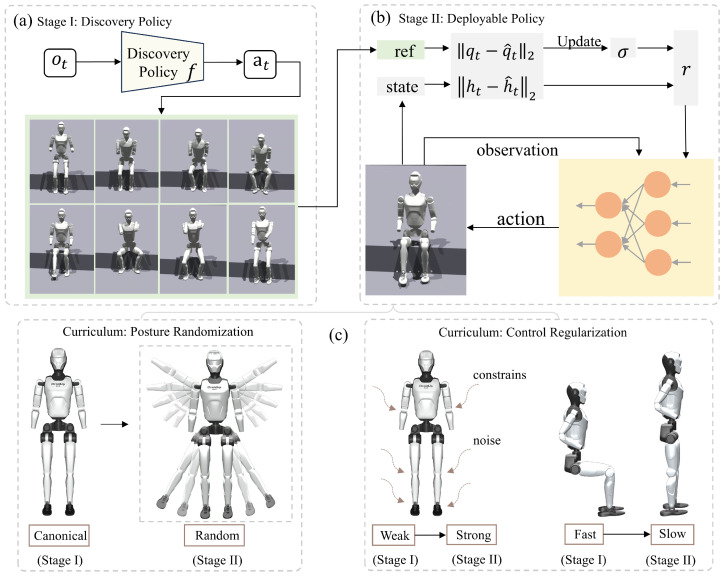
Overview of the two-stage RL approach for Sitting and Standing-Up Learning. (**a**) In the **first stage**, we learn a discovery policy *f* to identify a sit-up motion trajectory under minimal constraints. (**b**) In the **second stage**, the trajectory obtained in stage I is transformed into a deployable policy π that is robust and generalizable. This policy is trained by adaptively following a temporally slowed version of the discovered trajectory with strong control regularization, over varied terrains and initial configurations. (**c**) The two-stage training procedure establishes a curriculum: stage I emphasizes motion exploration in simplified settings (e.g., fixed initial poses, flat terrain, weak regularization, and noise), whereas stage II aims to acquire a safer and more practically deployable policy.

To tackle these issues, we introduce a **two-stage reinforcement learning (RL) framework**, as illustrated in Figure 2:

- **Stage 1: Exploration policy training.** In a simplified environment without noise or randomization, a policy is trained to explore feasible sit-stand motion sequences. By incorporating assistive forces during this exploration phase, the robot is guided towards discovering natural and coordinated motions. The primary objective of this stage is to address the sparsity of rewards and the challenge of action discovery.
- **Stage 2: Deployable policy training.** Building on the trajectories discovered in stage 1, we train a deployable policy that incorporates trajectory tracking, noise injection, and domain randomization. This stage improves the smoothness, robustness, and real-world applicability of the policy.

During training, we employ a full robot model that includes physical constraints such as body collisions and joint friction, making the simulation closely approximate the real system. In the second stage, various domain randomization strategies are introduced, including pose randomization to mitigate zero-point offsets in the real robot, as well as external force perturbations and center-of-mass variations. The resulting policy demonstrates high robustness and can be directly transferred from simulation to a real humanoid robot, achieving stable sit-to-stand control across multiple scenarios.

### 3.1. RL Training

We formulate the humanoid robot sit-to-stand and stand-to-sit control problem as a finite-horizon Markov Decision Process (MDP) [43], defined by the tuple M=〈S,A,T,R,γ〉. At each time step *t*, the agent (i.e., the robot) observes a state $s_{t}\in S$ from the environment and samples an action $a_{t}\in A$ according to its policy $\pi_{\theta }\left(\cdot |s_{t}\right)$. The environment then transitions to the next state $s_{t+1}∼T\left(\cdot |s_{t},a_{t}\right)$ and provides a reward signal $r_{t}\in R$.

To solve this MDP, we adopt reinforcement learning (RL) [43] methods. The objective is to learn an optimal policy $\pi_{\theta }$ that maximizes the expected cumulative reward $E_{\pi_{\theta }}\left[\sum_{t=0}^{T-1}\gamma^{t}r_{t}\right]$ over a episode of length *T*, For detailed notation, refer to Table A1.

Where γ∈[0,1] is the discount factor. The expected return is estimated by a value function (critic) $V_{\phi }$. In this work, we adopt Proximal Policy Optimization (PPO) [44] as our reinforcement learning algorithm due to its stability and efficiency in large-scale parallel training.

#### 3.1.1. State Space

We assume that proprioceptive information alone provides sufficient knowledge for controlling the stand-up behavior in the target environment. Therefore, we define the state to include the robot’s proprioceptive signals obtained from the inertial measurement unit (IMU) and joint encoders as follows:

$$s_{t}=\left[\omega_{t},r_{t},p_{t},{\dot{p}}_{t},a_{t-1},\beta \right]$$

Let $\omega_{t}\in R^{3}$ denote the angular velocity of the robot base, and $r_{t}$ and $p_{t}$ represent the roll and pitch angles, respectively. The joint positions and velocities are denoted by $p_{t}\in R^{19}$ and ${\dot{p}}_{t}\in R^{19}$, and the previous action is denoted by $a_{t-1}\in R^{19}$. A scalar β∈(0,1] is used to scale the output actions.

Given the strong contact characteristics of the stand-up task, we enhance the contact-awareness of the policy by implicitly incorporating contact information through the inclusion of the past five historical states in the policy input [15]. In both training phases, the observation input is denoted by $s_{t}$.

Kinetic Considerations: The state variables $p_{t}$ and ${\dot{p}}_{t}$ correspond to the generalized coordinates and generalized velocities in the robot’s Lagrangian dynamics.

#### 3.1.2. Reward

We present the set of reward functions used in stage I along with their detailed descriptions in Table 1.

$r_{\mathrm{head}\;\mathrm{height}}$: Encourages the robot’s head height to be close to a target height during sitting;

$r_{\mathrm{base}\;\mathrm{orien}}$: Encourages the robot to maintain an upward-facing posture, i.e., the projection of gravity on the base *z*-axis is close to 1;

$r_{\Delta \mathrm{base}\;\mathrm{height}}$: Encourages the robot to continuously lower its base height;

$r_{\mathrm{soft}\;\mathrm{body}\;\mathrm{symmetry}}$: Encourages symmetric motions across the body, which accelerates policy exploration.

$r_{\mathrm{hip}}\;\mathrm{roll}/\mathrm{yaw}\;\mathrm{deviation}$: It penalizes the large joint angle of hip roll/yaw joints.

$r_{\mathrm{knee}}\;\mathrm{deviation}$: It penalizes the large joint angle of knee joints.

$r_{\mathrm{foot}}\;\mathrm{distance}$: It penalizes a far distance between feet.

$r_{\mathrm{shank}}\;\mathrm{orientation}$: It encourages the left/right shank to be perpendicular to the ground.

Tracking task rewards:

$r_{\mathrm{tracking}}\;\mathrm{dof}\;\mathrm{position}$: Encourages the robot to track the reference joint trajectory;

$r_{\mathrm{termination}}$: Penalizes the robot for undesired early terminations during execution.

To accommodate the multi-stage nature of the sit-up task, we activate different reward terms according to the robot’s base height at each stage. Throughout training, all rewards adopt a unified Gaussian shaping formulation to ensure smooth gradient transitions and stable policy optimization. Specifically, we categorize the rewards into three groups: (1) task rewards that define the high-level task objectives; (2) regularization rewards that suppress abnormal joint configurations or excessive deviations; and (3) tracking rewards, activated in the second stage, that guide the robot to follow the reference motion. This design enables smooth transitions across stages and facilitates the progressive emergence of the desired sit-up behavior.

#### 3.1.3. Action Space

We employ a torque-based Proportional-Derivative (PD) controller to drive the robot. The action $a_{t}$ represents the offset between the current joint position and the desired target position for the next time step. The desired position for the PD controller is computed as:

(1) $$p_{t}^{d}=p_{t}+a_{t}$$

where each dimension of the action $a_{t}$ is constrained within the range [−1,1].

At time step *t*, the resulting joint torques are calculated as:

(2) $$\tau_{t}=K_{p}\cdot \left(p_{t}^{d}-p_{t}\right)-K_{d}\cdot {\dot{p}}_{t}$$

where $K_{p}$ and $K_{d}$ denote the stiffness and damping coefficients of the PD controller, respectively. The action space dimensionality |A| corresponds to the number of actuators in the robot. Although torque-based proportional control is known to be robust for whole-body control tasks, its performance inherently depends on the actuator limits and the nonlinearities of the robot’s dynamics, which may constrain the maximum achievable tracking accuracy.

### 3.2. Curriculum Learning

A two-stage policy learning approach is adopted through a curriculum-based training framework that gradually increases task complexity. Figure 2 depicts the first phase, in which we focus on motion exploration in a relatively simple environment, characterized by weak regularization, no terrain variation, a unified initial posture, and auxiliary assistance forces. This setting facilitates the emergence of recovery behaviors. The second stage aims to transform these behaviors into deployable and generalizable strategies. Specifically, the complexity of the training environment increases across several aspects from stage I to stage II:

**Posture randomization:** In stage I, training begins from standardized poses to accelerate the learning of recovery motions, such as using consistent seated and standing joint configurations. In stage II, posture randomization is introduced to improve the generalizability of the learned policy. Inspired by how infants use external support to develop motor skills, we design an environment-assisted mechanism to accelerate exploration. Specifically, an upward vertical force *F* is applied to the robot’s torso at the early phase of training, which is gradually reduced as the robot becomes capable of maintaining the target height on its own.

**Control regularization:** Stage I employs weak regularization on joint positions, velocities, and accelerations, promoting the discovery of effective standing-up behaviors. In contrast, Stage II progressively strengthens regularization according to the curriculum schedule, including increased penalties on action smoothness and joint torques, thereby encouraging the development of more deployable and stable control strategies.

**Environment randomization:** In stage I, the environment remains relatively fixed, such as using a uniform height for seated positions and disabling parameter randomization. In stage II, we introduce domain randomization and noise to better simulate real-world uncertainties and enhance the robustness of the learned policy.

### 3.3. Adaptive Motion Tracking

In the absence of strong regularization constraints, the policy learned in the first stage may result in extremely fast yet unsafe sit-up motions (less than 1 s), which are undesirable for real-world applications. To address this issue, the second stage focuses on learning safer and slower sit-up motions. Specifically, we interpolate the motions learned in the first stage to generate a more suitable reference trajectory. During training, the agent is encouraged to track this reference trajectory, and the tracking reward is formulated using an exponential function as follows:

(3) r(x)=exp(−x/σ)

Here, *x* represents the deviation from the reference trajectory, commonly quantified using the mean squared error (MSE) of variables such as joint angles. The parameter σ determines the acceptable error range and is referred to as the tracking coefficient. Unlike formulations based on negative errors, the exponential form is favored because it is bounded, contributing to training stability and offering a more intuitive way to adjust rewards.

Intuitively, if σ is considerably greater than the typical magnitude of *x*, the reward stays near 1 and exhibits low sensitivity to fluctuations in *x*. Conversely, when σ is too small, the reward approaches 0, also reducing its sensitivity to *x*. This highlights the importance of choosing an appropriate value for σ to ensure sufficient responsiveness of the reward, thereby improving tracking accuracy.

To identify an appropriate value for the tracking factor, we construct a simplified model of motion tracking and express the problem as a bi-level optimization task.

The rationale for this formulation is that the tracking factor σ should be chosen to minimize the cumulative tracking error along the reference trajectory resulting from the converged policy. In traditional manual tuning, this is commonly performed through an iterative procedure: an engineer selects a candidate value for σ, trains the policy, evaluates the outcome, and repeats this cycle until satisfactory performance is obtained.

For a given policy π, we can derive a sequence of expected tracking errors $x\in R_{+}^{N}$, where $x_{i}$ represents the anticipated tracking error at the *i*-th rollout step.

Instead of directly optimizing the policy, we treat the tracking error sequence *x* as the decision variable. This enables us to reformulate the motion tracking optimization problem as:

(4) $$\max_{x\in R_{+}^{N}}J_{\mathrm{in}}\left(x,\sigma \right)+R\left(x\right)$$

Here, the internal objective

(5) $$J_{\mathrm{in}}\left(x,\sigma \right)=\sum_{i=1}^{N}\exp\left(-x_{i}/\sigma \right)$$

represents the simplified cumulative reward corresponding to the tracking reward introduced in Equation (3), while R(x) accounts for additional contributions beyond $J_{\mathrm{in}}$, including environmental dynamics and other policy-specific objectives, such as auxiliary rewards.

The solution $x^{*}$ to Equation (4) denotes the error sequence generated by the optimal policy $\pi^{*}$. Our subsequent goal is to maximize the cumulative negative tracking error, formalized as the external objective:

(6) $$\max_{\sigma \in R^{+}}J_{\mathrm{ex}}\left(x^{*}\right),\;s.t.\;x^{*}\in \arg\max_{x\in R_{+}^{N}}J_{\mathrm{in}}\left(x,\sigma \right)+R\left(x\right)$$

Under a few additional technical assumptions, Equation (6) can be solved analytically, revealing that the optimal tracking factor $\sigma^{*}$ is equal to the mean value of the optimal tracking error sequence:

(7) $$\sigma^{*}=\left(\sum_{i=1}^{N}x_{i}^{*}\right)/N$$

## 4. Simulation Experiments

### 4.1. Task Setup

We evaluate the humanoid robot on two sit-to-stand related tasks:

1. Transition from a standing posture to a sitting posture.
2. Transition from a sitting posture to a standing posture.

The simulation experiments are conducted using the complete URDF model.

### 4.2. Metrics

The evaluation metrics for the sit-to-stand and stand-to-sit tasks of humanoid robots remain an open research problem. We propose the following metrics to assess task performance.

#### 4.2.1. Success Rate

A trial is considered successful if the robot’s center-of-mass (CoM) height reaches the target height and remains above that threshold for the remaining time steps, indicating that the robot has achieved a stable posture.

For the stand-up task, the robot is considered to have reached the target posture once it restores an upright configuration, where the base link is approximately aligned with the vertical axis and the legs are largely extended. The corresponding height condition is defined as:

base_height=1.0±0.02m.

For the sit-down task, the robot is considered to have reached a stable seated posture when the torso remains upright. Since the pelvis height is determined primarily by the chair height, the success condition is defined as:

base_height=(chairheight+0.13)±0.02m.

#### 4.2.2. Motion Smoothness

Beyond success rate, motion smoothness is an important metric for evaluating whether the sit-to-stand motion is continuous and safe. Smoothness is quantified by aggregating the angular changes of all joints between consecutive control steps over the entire episode. Specifically, for each joint, the difference in joint angles between successive time steps is computed, the absolute value is taken, and the results are summed over all joints and all time steps to yield a single smoothness score.

We use the following parameters: Action Jitter (rad/s^3^); DoF Position Jitter (rad/s^3^).

#### 4.2.3. Safety

We introduce two safety coefficients: the torque safety coefficient $S_{\mathrm{Torque}}$ and the DoF angle safety coefficient $S_{\mathrm{DoF}}$.

These coefficients measure whether the joint torques and joint angles exceed their respective physical limits. Maintaining these within safe bounds is essential, as excessive torque or joint angles may cause task failure or result in mechanical and motor damage.

(8) $$\begin{array}{cc} S_{\mathrm{Torque}}\; & =\frac{1}{TJ}\sum_{t=1}^{T}\sum_{j=1}^{J}⊮\;\left(\frac{|\tau_{t,j}|}{\tau_{j}^{\max}}\leq 1\right),\\ S_{\mathrm{DoF}}\; & =\frac{1}{TJ}\sum_{t=1}^{T}\sum_{j=1}^{J}⊮\;\left(\frac{|q_{t,j}|}{q_{j}^{\max}}\leq 1\right). \end{array}$$

$\tau_{t,j}$ and $q_{t,j}$ denote the applied torque and joint angle of the *j*-th joint at time step *t*, respectively.

$\tau_{j}^{\max}$ and $q_{j}^{\max}$ represent the maximum allowable torque and the range limit of the *j*-th joint.

*T* denotes the total number of time steps, and *J* is the total number of joints.

### 4.3. Baseline Methods

We compare our approach with the following baseline methods:

1. Single-stage training without considering torque/control constraints.
2. Single-stage training with torque/control constraints, but without the proposed two-stage training scheme.
3. Two-stage training without employing the tracking factor in the second stage.

Table 2 presents our experimental results.

(1). Ignoring Torque/Control Limits Leads to Non-deployable Policies.

Although policies trained without considering torque or control limits can also achieve relatively high task success rates, the performance in terms of smoothness and safety is substantially worse than that of our method. For instance, the joint velocities are nearly an order of magnitude higher (almost 10×) than those in our method. As a result, the learned actions are unstable and unsafe, making such policies infeasible for deployment on real robots.

(2). Considering Torque/Control Limits with Single-stage Training.

It is well recognized that incorporating control limits is essential for stability and safety in sim-to-real transfer. However, applying all torque and control constraints in a single-stage training setting prevents the policy from accomplishing the task, since strict regularization significantly hampers exploration. In contrast, our two-stage curriculum learning framework balances exploration in the first stage with stability and safety in the second stage, leading to policies that produce actions which are both natural and safe.

(3). Without Adaptive Tracking Factors in the Second Stage.

We compare our approach with methods that employ fixed tracking factors. The results show that using a fixed tracking factor leads to significant performance discrepancies between sitting-down and standing-up motions, failing to achieve optimal tracking in both cases. By comparison, our adaptive tracking mechanism reliably attains near-optimal performance for both motions, demonstrating the effectiveness of dynamically adjusting the tracking factor to accommodate different movement patterns.

**Figure 3:**
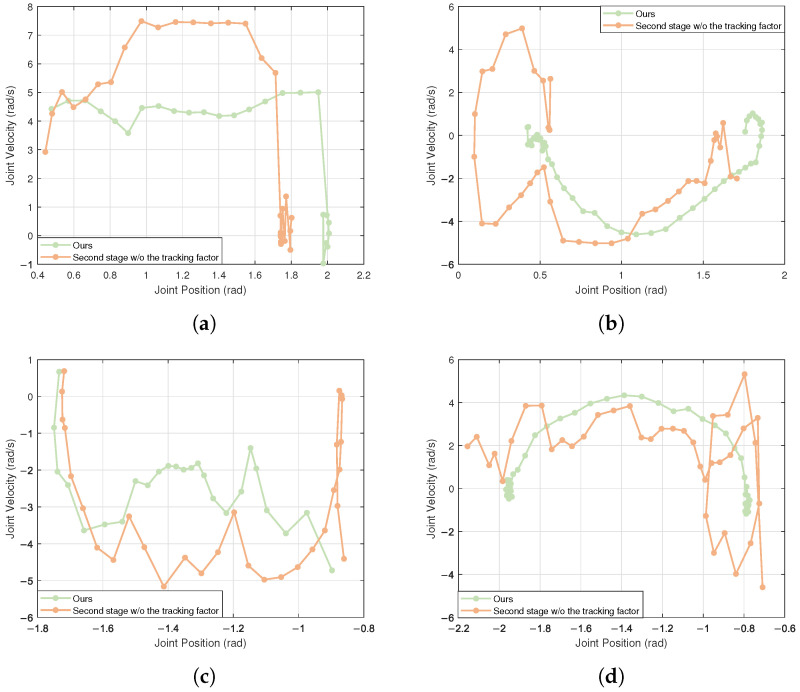
Stability comparison. (**a**,**c**) show the hip and knee joint phases during sitting down; (**b**,**d**) show the hip and knee joint phases during stand up.

To facilitate a more intuitive analysis of whether the joint angles and velocities change smoothly during the sit-down and stand-up processes of the robot, phase diagrams of joint position versus velocity were plotted. The most critical joints in this context are the hip pitch joint and the knee joint. As illustrated in Figure 3, our strategy demonstrates smoother velocity variations compared to the second stage without the tracking factor, with smaller oscillations at the end of the task, thereby resulting in more stable and natural movements.

Summary of baseline comparisons. The comparison of baseline methods highlights three key observations:

(1) ignoring torque and control limits results in non-deployable policies with unstable and unsafe actions;
(2) applying all torque and control limits in a single-stage training restricts exploration, preventing successful task completion;
(3) omitting adaptive tracking factors in the second stage leads to suboptimal tracking performance for both sit-down and stand-up motions.

Overall, our two-stage training framework, which incorporates control constraints and adaptive tracking factors, achieves high success rates while producing smooth, stable, and natural movements.

## 5. Real-World Results

We evaluate our policy on the X02 robot in the real world. Two main tasks are tested:

1. Transition from standing to sitting
2. Transition from sitting to standing

We compare our policy against Host and a high-performance ablation variant (without the tracking factor in Stage II). The policy provided by Host is based on a multi-critic architecture, which learns stand-up behaviors across diverse terrains. Their experiments also include the transition from sitting to standing, but it is achieved by training the robot to rise from a supine position on the platform, thereby covering the sit-to-stand case indirectly. Directly adopting this method causes the robot to lean backward significantly at the beginning, making the motion unstable.

**Figure 4:**
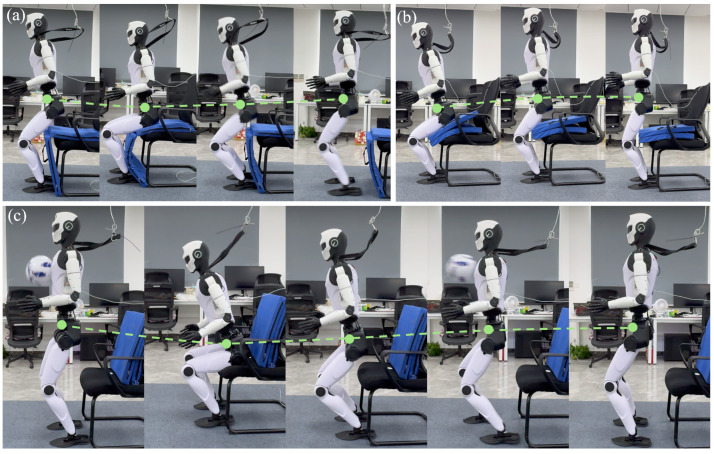
Real-world results. We evaluated our method in various real-world environments. (**a**,**b**) represent different terrain heights, and (**c**) represents external disturbances. The results demonstrate that our method can successfully complete the sitting and standing-up tasks. The dashed line indicates the trajectory of the robot’s center of mass.

Figure 4 and Table 3 present the experimental results. Overall, our approach outperforms both the Host baseline and the two-stage variant without the tracking factor. The table provides detailed quantitative metrics, including the number of trials, estimated contact impulses, success counts, and completion times. Our policy demonstrates strong robustness: it consistently performs sit-to-stand and stand-to-sit motions even under variations in environmental configurations. This robustness is largely attributed to environment randomization applied during curriculum learning. Moreover, the policy remains stable under significant external disturbances, completing the tasks smoothly due to the effective modulation provided by adaptive motion tracking.

Experimental environments: Base environment: Fabric seat + armrest, seat pitch −3°, dimensions 50 × 40 × 45 cm (L × W × H).

Terrain A: Base environment + polyester cushion, 40 × 40 × 5 cm;

Terrain B: Base environment + polyester cushion, 40 × 40 × 10 cm.

(1) Standing to Sitting

Since Host is mainly designed for stand-up behaviors, it fails to handle this task. Our policy also demonstrates higher stability and robustness compared to the variant without the tracking factor. In general, the sitting task is easier than the sit-to-stand task. Our policy achieves a success rate of 95% on this task.

(2) Sitting to Standing

The Host framework is capable of enabling the Unitree G1 robot to stand up from a chair. However, when applied to our robot, this process fails. This failure can be attributed to the fact that our robot lacks the roll joint in the ankle, which is part of the leg’s degrees of freedom in the G1. Additionally, our robot is 170 cm tall, which is higher than the G1, further increasing the difficulty of the task.

The variant without the tracking factor produces motions that are less smooth than those generated by the complete framework, as it cannot better reconcile trajectory tracking with the overall task requirements.

By contrast, our policy generates highly smooth motions, allowing seamless switching between sitting and standing. This also validates its strong adaptability to different initial states.

## 6. Limitations

Inheritance between Stage I and Stage II. There may be discrepancies between the actions explored in Stage II and those discovered in Stage I. This arises because Stage II reduces the action-constraining rewards while strengthening control regularization. To preserve action consistency, key motion-style rewards from Stage I can be retained in Stage II.

Transitions between Sitting and Standing. During the transition between sitting and standing, improper sitting placement can significantly compromise overall stability. If the robot sits either too close to or too far from the chair, its center of mass may fall into a configuration that is unfavorable for the subsequent standing motion, thereby requiring substantial postural compensation and increasing the likelihood of failure. To address this issue, we define a “safety region” based on the horizontal distance between the robot’s heels and the seat edge. Extensive experiments show that maintaining this distance within 0–15 cm yields the most stable sitting posture for initiating the standing motion. Distances below 0 cm result in overly deep seating, restricting forward momentum, whereas distances beyond 15 cm lead to shallow seating with insufficient support, increasing the risk of slipping. To further validate the effectiveness of this safety region, we conducted misalignment experiments involving lateral offsets of ±5–10 cm and yaw offsets of ±10–15° prior to sitting. The results indicate that as long as the heel-to-seat distance remains within the 0–15 cm interval, the robot maintains a success rate exceeding 90%, even under such perturbations. Once this distance falls outside the interval, however, the success rate drops to 40–60%. These findings confirm the practical necessity and robustness of the proposed safety region in real-world sitting-to-standing transitions.

Adaptability to Complex Terrains Our policy still lacks sufficient exploration for more complex scenarios, such as stepped chairs or uneven surfaces. This limitation stems from the difficulty of accurately modeling such cases in simulation. However, recent works such as Genesis [46], Mujoco Playground [47], and Roboverse [48] provide improved simulation capabilities for complex tasks involving perception and contact, which may help address these challenges.

## 7. Conclusions and Outlook

This paper proposes a two-stage reinforcement learning framework to address the control problem of sitting down and stand up for humanoid robots in real-world environments. In the first stage, feasible standing-up sequences are explored, while in the second stage, actions are refined by integrating trajectory tracking, adaptive modulation, and regularization constraints. This effectively alleviates the challenges of sparse rewards, complex contact patterns, and non-periodic behaviors. Both simulation and real-world experiments demonstrate that the proposed method enables the X02 robot to successfully perform sitting and standing tasks across different postures and terrains, exhibiting smoothness, stability, and robustness.

Despite these promising results, several limitations remain. Stage II actions may deviate from the key characteristics discovered in Stage I, the transition stability between sitting and standing requires further improvement, and adaptability to complex terrains has yet to be fully validated. Future work will consider incorporating style-preserving rewards, designing safety boundary constraints, and leveraging higher-fidelity simulation platforms as well as perception feedback to enhance policy generalization in diverse environments. Furthermore, potential extensions include applying the framework to multi-contact tasks (such as crawling or standing-up), and integrating learning-based perception modules to enable the policy to adapt in real time to dynamically changing environments.

Overall, this study provides a novel perspective for achieving natural and stable sitting and standing control in humanoid robots, and lays a methodological foundation for tackling other tasks involving complex contact patterns.

## Figures and Tables

**Table 1:** The reward functions used for learning the sitting and standing-up. In the first stage of training, no tracking rewards are added, while in the second stage, tracking rewards are incorporated. The regularization rewards are shared across both tasks. The function $f_{\mathrm{tol}}$ is a Gaussian-style function with saturation bounds; for more details, please refer to [45].

Term	Expression	Weight
**Regularization**		
Joint acceleration	$∥\ddot{p}{∥}^{2}$	$-2.5\times 10^{-7}$
Joint velocity	$∥\dot{p}{∥}_{2}^{2}$	$-1\times 10^{-4}$
Action rate	${∥a_{t}-a_{t-1}∥}^{2}$	$-1\times 10^{-2}$
Torques	${∥\tau ∥}^{2}$	$-2.5\times 10^{-6}$
Joint pos limits	$\sum_{i}\left[\left(p_{i}-p_{i}^{\mathrm{Lower}\;}\right)\cdot \mathrm{clip}\left(-\infty ,0\right)+\left(p_{i}-p_{i}^{\mathrm{Higher}\;}\right)\cdot \mathrm{clip}\left(0,\infty \right)\right]$	$-1\times 10^{-2}$
**Sit Down**		
Head height	$f_{\mathrm{tol}\;}\left(h_{\mathrm{head}\;},\left[1,\infty \right],1,0.1\right)$	1
Base orientation	$f_{\mathrm{tol}\;}\left(-\theta_{\mathrm{base}\;}^{z},\left[0.99,\infty \right],1,0.05\right)$	1
Δ base height	$⊮\left(h_{t}^{\mathrm{base}}<h_{t-1}^{\mathrm{base}}\right)$	1
Soft body symmetry	$\exp(p^{l}-p^{r})$	1
**Stand Up**		
Head height	$f_{\mathrm{tol}\;}\left(h_{\mathrm{head}\;},\left[h_{\mathrm{target}},h_{\mathrm{target}}+0.1\right],1,0.1\right)$	1
Base orientation	$f_{\mathrm{tol}\;}\left(-\theta_{\mathrm{base}\;}^{z},\left[0.99,\infty \right],1,0.05\right)$	1
Hip roll/yaw deviation	$⊮\left(\max\left(\left|q_{\mathrm{hip}\;}^{1,r}\right|\right)>0.7\right)|⊮\left(\min\left(\left|q_{\mathrm{hip}\;}^{1,r}\right|\right)>0.5\right)$	−10/−10
Knee deviation	$⊮\left(\min\left(\left|q_{\mathrm{knee}\;}^{1,r}\right|\right)<-2.1\right)|⊮\left(\max\left(\left|q_{\mathrm{knee}\;}^{1,r}\right|\right)>-0.06\right)$	−10
Foot distance	${∥q_{\mathrm{feet}\;}^{l}-q_{\mathrm{feet}\;}^{r}∥}^{2}>0.9$	−10
Shank orientation	$f_{\mathrm{tol}\;}\left(\mathrm{mean}\left(\theta_{\mathrm{shank}\;}^{1,r}\left[2\right]\right),\left[0.8,\infty \right],1,0.1\right)\times ⊮\left(h_{\mathrm{base}\;}>H_{\mathrm{stage}\;1}\right)$	10
**Tracking**		
Tracking DoF position	$\exp\left(-\frac{{\left(d_{t}-d_{t}^{\mathrm{target}}\right)}^{2}}{4}\right)$	8
Termination	${⊮}_{\mathrm{termination}}$	−50

**Table 2:** Simulation results.

	Task			Smoothness				Safety	
	Success	Task Metric		Action Jitter	DoF Pos Jitter	Energy		$S_{0.8,0.5}^{\mathrm{Torque}}$	$S_{0.8,0.5}^{\mathrm{DoF}}$
**(a) Standing posture to a sitting posture**									
Single-stage w/o constraints	82.72 ± 0.54	0.67 ± 0.00		13.39 ± 0.01	0.71 ± 0.00	1210.19 ± 1.26		0.72 ± $3.10\times 10^{-4}$	0.73 ± $1.39\times 10^{-4}$
Single-stage w/constraints	50.32 ± 0.25	0.65 ± 0.00		5.70 ± 0.18	0.48 ± 0.00	121.22 ± 0.67		0.57 ± $1.36\times 10^{-3}$	0.55 ± $5.56\times 10^{-4}$
Two-stage w/o tracking factor	98.56 ± 0.42	0.67 ± 0.05		0.90 ± 0.01	0.14 ± 0.00	104.14 ± 0.64		0.92 ± $3.41\times 10^{-5}$	0.74 ± $2.34\times 10^{-5}$
Ours	**99.8 ± 0.50**	0.68 ± 0.04		**0.55 ± 0.01**	**0.10 ± 0.00**	**91.47 ± 0.32**		**0.93 ± $1.54\times 10^{-5}$**	**0.79 ± $4.25\times 10^{-5}$**
**(b) Sitting posture to a standing posture**									
Single-stage w/o constraints	50.62 ± 0.34	1.55 ± 0.00		15.39 ± 0.02	0.68 ± 0.00	1650.19 ± 2.26		0.52 ± $3.52\times 10^{-4}$	0.51 ± $3.39\times 10^{-4}$
Single-stage w/constraints	24.82 ± 0.34	0.83 ± 0.01		6.70 ± 0.12	0.41 ± 0.02	151.22 ± 5.67		0.82 ± $2.16\times 10^{-3}$	0.68 ± $6.75\times 10^{-4}$
Two-stage w/o tracking factor	93.95 ± 0.32	1.00 ± 0.00		0.80 ± 0.03	0.20 ± 0.00	98.13 ± 0.46		0.88 ± $1.14\times 10^{-5}$	0.69 ± $3.42\times 10^{-5}$
Ours	**99.5 ± 0.07**	**1.58 ± 0.02**		**0.56 ± 0.50**	**0.10 ± 0.30**	**92.52 ± 0.46**		**0.93 ± $3.23\times 10^{-5}$**	**0.78 ± $2.45\times 10^{-5}$**

**Table 3:** Real-world experimental results across different environments. Contact impulse refers to the estimated impulse applied to the robot’s body during external interactions (0–43 N). Completion time is reported as mean ± standard deviation.

Task	Environment	Trials	Contact Impulse (N)	Success	Completion Time (s)
Sit-down	Base environment	20/10	0/37 ± 5	18/7	3.36 ± 0.30
	Terrain A	20/10	0/37 ± 5	18/9	3.17 ± 0.20
	Terrain B	20/10	0/37 ± 5	19/9	3.06 ± 0.20
Stand-up	Base environment	20/10	0/37 ± 5	16/7	2.12 ± 0.20
	Terrain A	20/10	0/37 ± 5	17/8	1.95 ± 0.20
	Terrain B	20/10	0/37 ± 5	18/9	1.86 ± 0.20

## Data Availability

The original contributions presented in this study are included in the article. Further inquiries can be directed to the corresponding author.

## Appendix A

**Table A1:** Abbreviations glossary for the Markov decision process.

Symbol	Description
S	State space of the robot
$s_{t}$	State observed at time step *t*
$s_{t+1}$	Successor state after executing $a_{t}$
A	Action space of the robot
$a_{t}$	Action executed at time step *t*
T	State transition function
R	Reward function
γ	Discount factor
$\pi_{\theta }$	Policy parameterized by θ
$r_{t}$	Reward received at time step *t*
*t*	Discrete time step index
M	MDP tuple
θ	Parameters of the policy
